# Alterations of lymph nodes evaluation after colon cancer resection: patient and tumor heterogeneity should be taken into consideration

**DOI:** 10.18632/oncotarget.11633

**Published:** 2016-08-26

**Authors:** Xu Guan, Wei Chen, Shuai Li, Zheng Jiang, Zheng Liu, Zhixun Zhao, Song Wang, Ming Yang, Xishan Wang

**Affiliations:** ^1^ Department of Colorectal Surgery, The Second Affiliated Hospital of Harbin Medical University, Harbin, China; ^2^ Department of Colorectal Surgery, Cancer Institute & Hospital, Chinese Academy of Medical Sciences, Peking Union Medical College, Beijing, China; ^3^ Follow Up Center, The Second Affiliated Hospital of Harbin Medical University, Harbin, China; ^4^ Department of Anesthesiology, The Third Affiliated Hospital of Harbin Medical University, Harbin, China

**Keywords:** colon cancer, lymph nodes, SEER, tumor features, patient features

## Abstract

Despite the adequacy of nodal evaluation was gradually improved for colon cancer (CC), rare attention has been paid for the effect of patient and tumor heterogeneity on nodal evaluation. We identified 109902 CC patients in stage I-III from Surveillance, Epidemiology, and End-Results (SEER) database during 2004-2013. The lymph nodes evaluations were separately assessed in different patient- and tumor-related features, including gender, age, T stage, histology, tumor differentiation, tumor size and tumor location. The 5-year cancer specific survival (CSS) was calculated with Kaplan-Meier method, log-rank tests were used to compare the differences of CSS in patients with ≥12 and <12 lymph nodes examined. Here, we identified features including gender, age, T stage, tumor differentiation, tumor size and location were independently associated with the median number of lymph node, the rate of ≥12 lymph nodes and the rate of node positivity of CC patients. We then divided CC patients into 29 subgroups according to different patient- and tumor-related features. The median number of lymph node presented a large variance from 12 to 24, the rate of ≥12 lymph nodes increased from 53.2% to 91.2% under the combined effect of patient and tumor heterogeneity. Furthermore, the positive association between increased lymph nodes count and improved survival couldn't be observed in 8261 CC patients with the effect of this heterogeneity. In conclusion, the tumor and patient heterogeneity lead to large alterations of nodal evaluation; we should pay more attention to this effect in clinical practice.

## INTRODUCTION

The accurate tumor staging of colon cancer (CC) is closely based on adequate regional lymph node examined. Accumulating evidences have demonstrated that sufficient lymph nodes examined were necessary because of the association with lymph node positivity, the decision of postoperative chemotherapy and improved long-term outcome [1–4]. Consequently, The Union of International Cancer Control (UICC) and American Joint Committee on Cancer (AJCC) guidelines recommends that evaluation of at least 12 lymph nodes during CC resection, which has been considered as a quality indicator for CC care [5, 6].

Despite the adequacy of nodal evaluation was gradually improved for CC, the disparity in nodal examination still begs the question of whether the 12-node measure should be the optimal threshold for all CC patients. It was well recognized that the number of lymph node examined was highly influenced by several factors. Surgical strategy usually contributed a lot to the variability of node evaluation, the larger extent of the colectomy by surgeons could directly increases the number of lymph nodes examined [7, 8]. Furthermore, the adequacy of pathological evaluation by pathologists may also affect the number of lymph nodes examined [9]. Several pathological examination techniques, such as lymph node-revealing solution and fat clearance with alcohol and xylene, have all been deemed as the improving lymph node assessment [10]. In addition to surgical and pathological variance, patient and tumor heterogeneity also take nonnegligible effect in affecting lymph nodes examination and further treatment planning for CC patients, which suggest that the application of 12-node measure should fully consider the combined effect of the tumor- and patient-related features.

The aims of this observational study were to establish for the first time to evaluate the effect of patient and tumor heterogeneity on nodal evaluation in a large-scale national cohort study. In this work, we firstly identify the patient- and tumor-related features that contribute to the variance of lymph nodes evaluation. Secondly, we evaluate the combined effect of the patient and tumor heterogeneity on nodal evaluation. Thirdly, we assess whether this heterogeneity influence the positive association between lymph nodes evaluation and the long-term survival.

## RESULTS

### Patient characteristics

From the SEER database, we totally identified 109902 CC patients including 56972 female and 52930 male. Patients aged 60-79 were accounted for 50.1% and patients aged 20-39 were only accounted for 2.2%. The proportion of patients in stage I was 23.3%, which was obviously smaller than patients in stage II (39.1%) and stage III (37.6%). 60.9% of patients had T3 primary tumor, the tumor size in 54.0% of patients was 2-5cm, and 71.6% was grade II. Additionally, only 10.7% of patients were diagnosed with mucous cancer, compared with 88.7% of adenocarcinoma. 37.5% underwent segmental resection and 60.0% underwent hemicolectomy. 76.3% of patients examined more than 12 lymph nodes. The detailed characteristic information was shown in Table 1.

**Table 1:** Characteristics of all CC patients in the SEER database: 2004-2013

Characteristics	No. of Patients	Percent
Gender		
Female	56972	51.8%
Male	52930	48.2%
Age		
20-39	2425	2.2%
40-59	26008	23.7%
60-79	55087	50.1%
≥80	26382	24.0%
Calendar year		
2004-2008	56167	51.1%
2009-2013	53735	48.9%
Stage		
Stage I	25606	23.3%
Stage II	42967	39.1%
Stage III	41329	37.6%
T stage		
T1	11524	10.5%
T2	19073	17.4%
T3	66929	60.9%
T4	12376	11.2%
Tumor size (cm)		
0-2	14797	13.5%
2-5	59363	54.0%
≥5	35742	32.5%
Grade		
Grade I	9789	8.9%
Grade II	78739	71.7%
Grade III	19018	17.3%
Grade IV	2356	2.1%
Histology		
Adenocarcinoma	97484	88.7%
Mucous cancer	11725	10.7%
Others	693	0.6%
Location		
Right colon	68638	62.5%
Left colon	41264	37.5%
Treatment		
Segmental resection	41217	37.5%
Hemicolectomy	65979	60.0%
Others	2706	2.5%
Lymph nodes examined		
<12 lymph nodes	26068	23.7%
≥12 lymph nodes	83834	76.3%

### Comparisons of median number of lymph nodes examined

**Figure 1:**
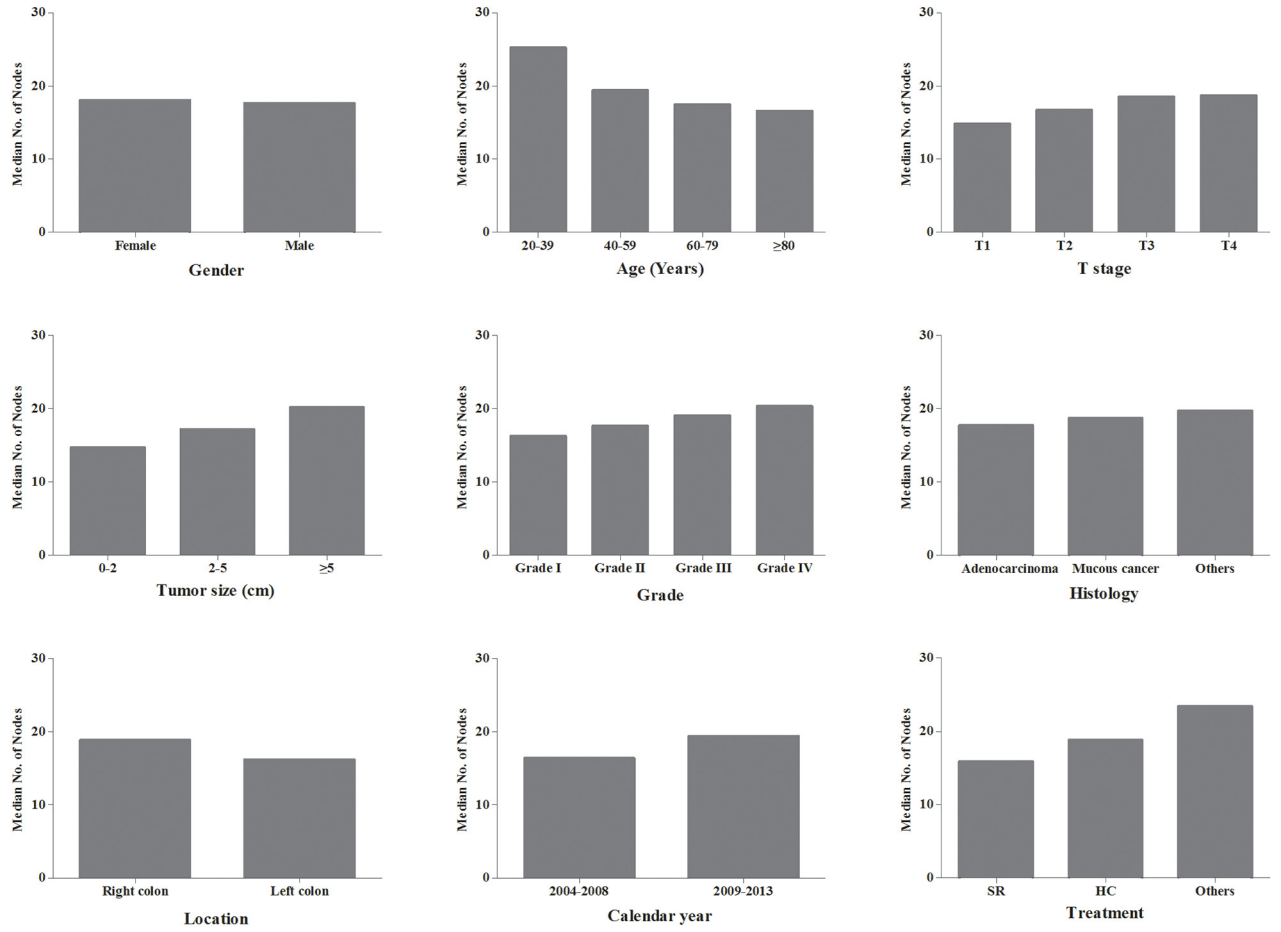
The median number of lymph nodes examined according to different patient- or tumor-related features HC: hemicolectomy; SR: segmental resection.

The median number of lymph nodes was 17.8 in all CC patients. We compared the median number of lymph nodes among different features (Figure 1). The female patients examined 18.0 lymph nodes, which was more than male patients (17.6) (P<0.001). The number of lymph nodes gradually decreased from 25.2 to 16.5 with age increasing (P<0.001). With the deeper tumor invasion, more lymph nodes could be examined from 14.8 to 18.7 (P<0.001). With tumor size increased, more lymph nodes were examined (P<0.001). Patients in grade III and IV examined more lymph nodes compared with the patients in grade I and II (P<0.001). Adenocarcinoma examined 17.7 lymph nodes, which was less than other histology types. Right-side CC examined 18.8 nodes, whereas left-side CC examined 16.2 (P<0.001). Hemicolectomy had a harvest of 18.8 lymph nodes, segmental resection only examined 15.9 lymph nodes (P<0.001).

### Comparisons of the rate of ≥12 lymph nodes and the rate of node positivity

**Figure 2:**
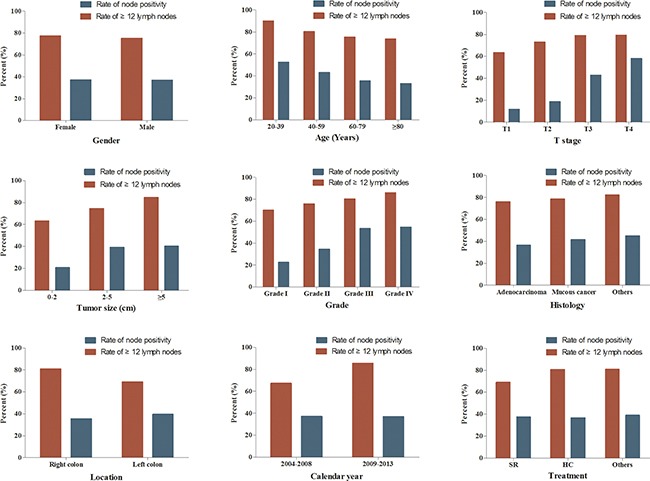
The rate of ≥12 lymph nodes examined and the rate of node positivity according to different patient- or tumor-related features HC: hemicolectomy; SR: segmental resection.

Then, the rate of ≥12 lymph nodes and the rate of node positivity were compared among different features (Figure 2). With the age increased, the rate of ≥12 lymph nodes and the rate of node positivity were obviously decreased (P<0.001). The rates of ≥12 lymph nodes and the rate of node positivity increased with higher T stage, poorer tumor differentiation and larger tumor size (P<0.001). Compared with left-side CC patients, right-side CC patients were more frequently examined with ≥12 lymph nodes but less frequently examined positive node (P<0.001). The rate of ≥12 lymph nodes for patients underwent hemicolectomy was 80.6%, which was significantly higher than segmental resection (69.0%) (P<0.001). In contrast, the rate of node positivity for patients underwent hemicolectomy was only 36.5%, which was unexpectedly lower than segmental resection (37.4%) (P<0.001).

In addition, multivariate analysis showed that female, younger patients, higher T stage, larger tumor size, poorer tumor differentiation, mucous cancer, right-side CC and hemicolectomy were independently associated with ≥12 lymph nodes examined (Table 2).

**Table 2:** Multivariate logistic regression analysis of obtaining ≥12 lymph nodes in CC patients

		Odds ratio	95% CI	P
Gender	Female	1		<0.001
	Male	0.880	0.854-0.906	
Age	20-39	1		<0.001
	40-59	0.516	0.448-0.594	
	60-79	0.353	0.307-0.406	
	≥80	0.276	0.240-0.317	
T stage	T1	1		<0.001
	T2	1.367	1.290-1.448	
	T3	1.415	1.297-1.544	
	T4	1.133	1.029-1.248	
Tumor size (cm)	0-2	1		<0.001
	2-5	1.357	1.286-1.431	
	≥5	2.279	2.149-2.417	
Grade	Grade I	1		<0.001
	Grade II	1.147	1.091-1.206	
	Grade III	1.205	1.133-1.282	
	Grade IV	1.379	1.208-1.574	
Histology	Adenocarcinoma	1		<0.001
	Mucous cancer	0.898	0.854-0.943	
	Others	0.926	0.748-1.146	
Location	Right colon	1		<0.001
	Left colon	0.607	0.586-0.629	
Treatment	Segmental resection	1		<0.001
	Hemicolectomy	1.498	1.447-1.552	
	Others	1.640	1.480-1.818	

### Effect of patient and tumor heterogeneity on nodal evaluation

**Figure 3:**
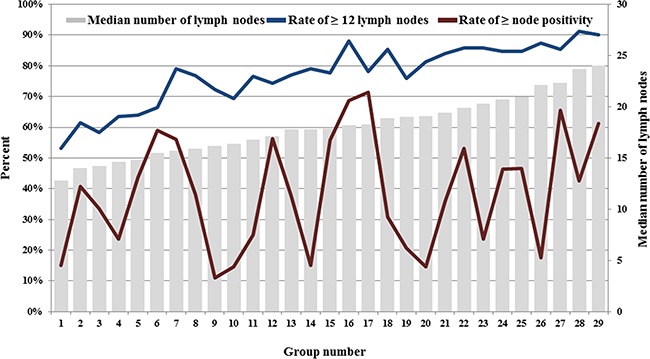
The variances of nodal evaluation of CC patients in 29 subgroups

To further observe the effect of tumor- and patient-related feature on nodal evaluation, we then divided patient into 32 subgroups based on five categorical variables, including age (<60 and ≥60), T stage (T1/T2 and T3/T4), tumor grade (grade 1/2 and grade 3/4), tumor location (right colon and left colon), tumor size (<5cm and ≥5cm). Three subgroups including less than 100 patients were ignored in further analysis to confirm the statistical accuracy. We reorder the subgroup number according to the median number of lymph nodes examined. The detailed subgroup information was listed in Table 3. The median number of lymph nodes, the rate of ≥12 lymph nodes and the rate of node positivity were separately calculated for each subgroup. With the effect of patient- and tumor-related features, the median number of lymph node increased from 12 to 24 (Figure 3). The large variation of rate of ≥12 lymph nodes was also found among those 29 subgroups, increasing from 53.2% to 91.2%, the trend was similar with the median number of lymph node examined. However, the rate of node positivity changed within a large variation in different subgroups, and trend of the rate was not related to the number of lymph nodes examined. This result showed a large variance of nodal evaluation under the effect of patient and tumor heterogeneity, suggesting that 12-node measure may not be equally required for all CC patients in the consideration of this large variation among different subgroups. The effect of this heterogeneity played a crucial role in lymph nodes evaluation.

**Table 3:** Patient- or tumor-related features of CC patients in 29 subgroups

Group number	Age (Years)	T stage	Tumor grade	Location	Tumor size (cm)	No. of patients	Median of nodes
1	≥60	T1-T2	Grade I-II	Left	0-5	6180	12.8
2	20-60	T1-T2	Grade III-IV	Left	0-5	332	14.0
3	≥60	T1-T2	Grade III-IV	Left	0-5	536	14.2
4	20-60	T1-T2	Grade I-II	Left	0-5	3284	14.6
5	≥60	T3-T4	Grade I-II	Left	0-5	9836	14.8
6	≥60	T3-T4	Grade III-IV	Left	0-5	1645	15.5
7	≥60	T3-T4	Grade III-IV	Right	0-5	4229	15.7
8	≥60	T3-T4	Grade I-II	Right	0-5	14898	15.9
9	≥60	T1-T2	Grade I-II	Right	0-5	11268	16.2
10	≥60	T1-T2	Grade I-II	Left	≥5	832	16.4
11	≥60	T1-T2	Grade III-IV	Right	0-5	1391	16.8
12	20-60	T3-T4	Grade I-II	Left	0-5	4789	17.1
13	≥60	T1-T2	Grade I-II	Right	≥5	2342	17.8
14	≥60	T3-T4	Grade I-II	Left	≥5	6512	17.8
15	≥60	T3-T4	Grade III-IV	Left	≥5	1313	17.9
16	20-60	T3-T4	Grade III-IV	Right	0-5	919	18.2
17	20-60	T3-T4	Grade III-IV	Left	0-5	881	18.3
18	20-60	T1-T2	Grade III-IV	Right	0-5	259	18.9
19	20-60	T1-T2	Grade I-II	Left	≥5	475	19.0
20	20-60	T1-T2	Grade I-II	Right	0-5	2501	19.1
21	≥60	T3-T4	Grade I-II	Right	≥5	13284	19.4
22	≥60	T3-T4	Grade III-IV	Right	≥5	6758	19.9
23	≥60	T1-T2	Grade III-IV	Right	≥5	389	20.3
24	20-60	T3-T4	Grade I-II	Left	≥5	3756	20.7
25	20-60	T3-T4	Grade I-II	Right	0-5	3564	21.0
26	20-60	T1-T2	Grade I-II	Right	≥5	633	22.1
27	20-60	T3-T4	Grade III-IV	Left	≥5	799	22.3
28	20-60	T3-T4	Grade I-II	Right	≥5	4374	23.7
29	20-60	T3-T4	Grade III-IV	Right	≥5	1748	24.0

### Effect of patient and tumor heterogeneity on the association between the nodal evaluation and 5-year CSS

**Figure 4:**
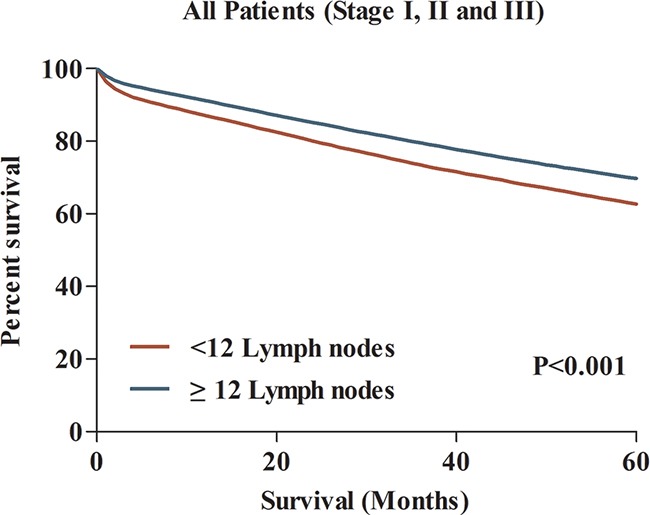
The comparison of 5-year CCS between patients with <12 and ≥12 lymph nodes examined

**Figure 5:**
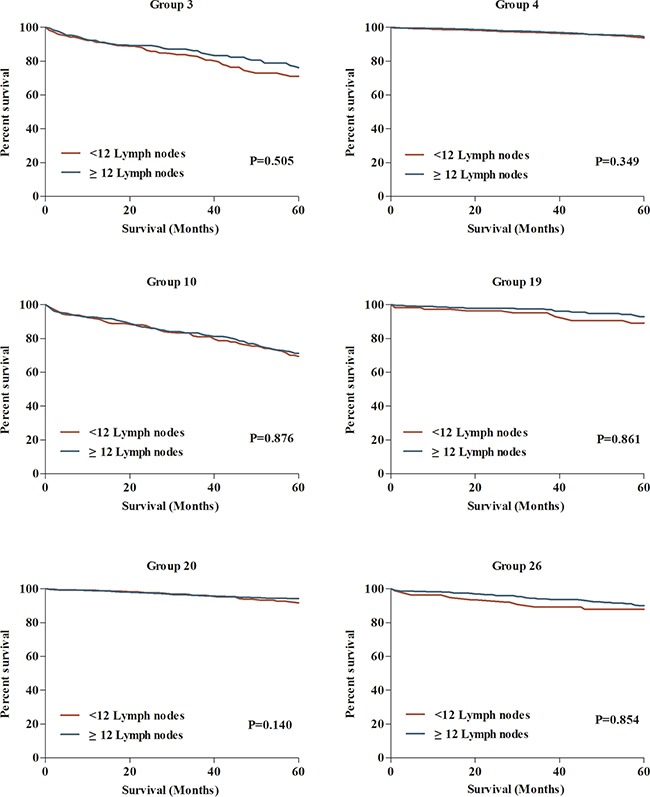
The comparisons of 5-year CCS between patients with <12 and ≥12 lymph nodes examined in 6 subgroups

Despite adequate lymph node evaluation lead to improved prognosis for CC patients, the effect of patient and tumor heterogeneity on the association between nodal evaluation and survival was unclear. We compared the difference of 5-year CSS between patient with≥12 and <12 lymph nodes in 29 subgroups. Taken as a whole, the 5-year CSS for patients with≥12 lymph nodes was 70.0%, which was significantly higher than patient with <12 lymph nodes (62.7%) (Figure 4). Interestingly, in separate analysis of 29 subgroups, we failed to observe the significant survival difference in subgroups of 3, 4, 10, 19, 20 and 26, totally including 8261 CC patients (Figure 5). This result might imply that despite increased lymph node yields lead to improved survival for CC patients, this association might not exist for some patients in the consideration of patient and tumor heterogeneity.

## DISSCUSION

The adequacy of the surgical resection and pathology examination was considered as potentially modifiable influential features for the completeness of lymph node assessment. However, the effect of patient- and tumor-related features also plays a nonnegligible role in nodal evaluation, which could not be altered by surgeons and pathologist. In this work, we found that the median number of lymph nodes changed within a large variation from 12 to 24, the rate of ≥12 lymph nodes was also found increasing from 53.2% to 91.2% in different subgroups. These large variances were mostly attributed to the patient and tumor heterogeneity.

**Figure 6:**
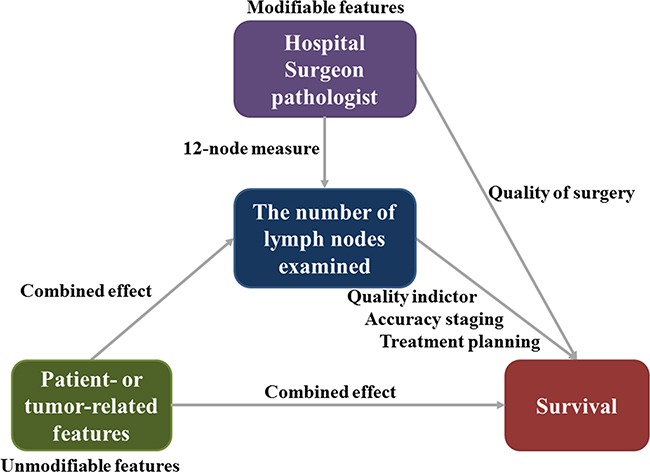
The effect of modifiable and unmodifiable features on the number of lymph nodes examined and survival in CC patients

In this study, we identified seven patient- or tumor-related features that contributed to the large variation of lymph node examined, including gender, age, tumor location, tumor differentiation, tumor invasion, tumor size and histology. These features were presented with a combined effect on the lymph node evaluation (Figure 6); they were associated with inherent differences that should be considered when comparing different patient populations. As we known, adjuvant chemotherapy is regularly recommended for selected stage II CC patients who have a harvest of <12 lymph nodes, which is considered as inadequate resection. The patient and tumor heterogeneity might indirectly determine the adjuvant chemotherapy according to variance of lymph node evaluation (Figure 6). For example, the patients with grade IV, aged 20-40, larger tumor size and right-side CC positively associated with the increasing number of lymph nodes evaluation, which could make them easy to obtain ≥12 lymph nodes and to avoid adjuvant chemotherapy. In contrast, it is hard to examine ≥12 lymph nodes for patient with grade I, aged >60, small tumor size and left-side CC, which could lead to increased probability of getting adjuvant chemotherapy for them. Thus, the effect of patient- or tumor-related features may be account for the change of prognosis for some stage II CC patients based on the number of lymph nodes evaluated. Of course, this disparity in nodal examination also begs the question of whether 12-nodes measure is appropriate for all CC patients.

The 12-node measure is currently applied as a clinical eligibility criterion for both lymph node resection and pathological examination for CC [11], and it emphasized the critical role of both extent of lymph node retrieval and the accuracy of nodal evaluation, not only regarding to staging accuracy, but also planning further treatment and determining prognosis. Due to the widespread application of the 12-node measure, an increasing number of lymph node was observed in previous studies [4, 12–15]. In our study, we found the median number of lymph node examined was 16.4 during the period of 2004 to 2008, which was obviously increased to 19.4 in the period of 2009-2013. However, the rate of node positivity conversely decreased from 37.1% to 36.8%. This result showed increasing lymph nodes evaluation was not contribute to higher rate of node positivity and improved staging accuracy. The quality of surgical resection plays a critical role in lymph nodes evaluation. An adequate specimen is composed of the segment of bowel containing the tumor, its associated mesentery and complete resection of draining lymph node of the primary tumor [8, 16, 17]. Here, we found that hemicolectomy were associated with higher number of lymph nodes examined compared with segmental resection for CC (HC vs SR: 18.8 vs 15.9), but this larger resection manner could not lead to higher rate of positive node (HC vs SR: 36.5% vs 37.4%). Good performance by surgeons and pathologists give rise to increased lymph node yields, but the non-modifiable features, such as patient- and tumor-features, could also influence the rate of node positivity and the accuracy of staging (Figure 6). It also implied that techniques might increase the lymph node count, but may not change the underlying nature of the disease.

Adequate lymph node evaluation for cancer involvement is crucial for prognosis of CC patients. Numerous studies have suggested that an adequate lymph node evaluation contributed to increased long-term survival [2, 18–21]. One systematic review of 17 studies from 9 countries, including five population-based observational studies, two multicenter randomized trials of adjuvant chemotherapy for CC, and 10 single-institution retrospective cohort studies, have suggested that the number of lymph nodes evaluated after surgical resection was positively associated with survival of patients with stage II and stage III CC. Despite heterogeneity in cutpoint numbers of lymph nodes evaluated, 4 of 6 studies concluded that increased survival of patients with stage III CC was associated with increased numbers of lymph nodes evaluated. 16 of 17 studies with data from stage II patients also showed a positive association with survival among patients with stage II CC [18]. In our study, the results also showed that a 7% higher 5-year CSS for all patients with ≥ 12 lymph nodes compared with <12 lymph nodes. In the consideration of this result, we need to distinguish this association from causality. Increased lymph node yields show an association with survival, but do not cause it. It is established that lymph node yields are multifactorial, influenced by a combination of not only surgical, pathological factors, but also patient- and tumor-related features [22, 23]. Accordingly, with the combined effect of patient and tumor heterogeneity, the positive association between the increased number of lymph nodes count and the improved survival could not be observed in some of CC patients.

Although the strengths of this work including large sample size, many concerned issues should be carefully explained. Firstly, tumor heterogeneity may not only include clinical and pathological features, but also involve molecular characteristics of cancer. However, the influence of molecular characteristics on lymph node evaluation is still unclear, and the SEER database lacked molecular information of cancer patients. Therefore, this work could not provide the effect of tumor molecular feature on lymph node evaluation of CC patients. Secondly, the SEER database collected cancer data from population-based cancer registries, which lead to large variations of pathologic techniques for detecting lymph nodes from 2004 to 2013. Although the pathologic techniques could influence lymph node staging in CC patients, we failed to obtain the related information and perform analyses in this large population-based study. Thirdly, it is impossible to provide a fixed node number to meet all variations of lymph node evaluation that caused by patient- and tumor-related features. However, an appropriate node number must be applied as the standard requirements for pathologic examination and quality of surgery in current clinical practice. At present, the 12-node measure may be considered as one feasible solution for this complex issue.

In conclusion, the lymph node evaluation of CC patients was closely associated with the effect of patient and tumor heterogeneity. The indicator of 12-node measure does not only reflect the quality of surgery and pathological evaluation, but also involve this heterogeneity. It is necessary to pay more attention to the effect of tumor and patient heterogeneity on nodal evaluation and treatment planning of CC patients.

## MATERIALS AND METHODS

### Data resources

We extracted cancer data from the Surveillance, Epidemiology, and End Results (SEER) database [24]. The SEER collected and published the cancer incidence, treatment and survival from 17 population-based cancer registries, which covered approximately 28 percent of the US population. The SEER database is considered to be the representative of the US population as a whole. The SEER database is an openly accessed database, cancer cases and population information could be obtained from the SEER. Data collected from the SEER database do not require informed patient consent, because they were anonymized and de-identified prior to release. We have got permission to access the cancer data from the SEER database by National Cancer Institute, and the reference number was 10249-Nov2015. This study was approved by the Second Affiliated Hospital of Harbin Medical University institutional review board.

### Study population

We identified patients older than 20 years who were diagnosed their first invasive CC in stage I to stage III from January 1, 2004 to December 31, 2013. The tumor staging was conducted according to the American Joint Commission on Cancer (AJCC TNM) (6th edition) staging system. Patients included in this study should undergo curative resection of CC as the first course of therapy, which were more available and accurate for the lymph node evaluation. Excluded criteria included patients who dead due to other causes, patients with an unknown number of nodes examined, patients who received preoperative radiotherapy and patients who underwent a local procedure or total colectomies.

### Patient- or tumor-related features

Patient features (gender, age at diagnosis), tumor features (AJCC stage, T stage, histology, grade, tumor size, tumor location, number of lymph nodes examined), year of diagnosis and surgical strategy were included in this study. The patients were divided into four age subgroups: 20-39 years, 40-59 years, 60-79 years and ≥80 years. Tumor size was classified as three subgroups: 0-2 cm, 2-5 cm and ≥5 cm. Histology included adenocarcinoma, mucous cancer and others. Tumor location included right-side CC and left-side CC, right-side CC was located proximal to the splenic colonic flexure, left-side CC from the splenic colonic flexure to the rectosigmoid junction. Surgical strategy was categorized as segmental resection, hemicolectomy and other procedures. Examining ≥12 lymph nodes was considered as adequate nodal evaluation, <12 lymph nodes was defined as poor node retrieval.

### Statistical analysis

The differences of lymph nodes evaluations were compared among each of patient- or tumor-related features in three ways: 1) Median number of lymph node examined. 2) Rate of ≥12 lymph nodes examined. 3) Rate of node positivity. All categorical variables were compared between groups using χ^2^ test. The logistic regression model was performed to estimate the factors that associated with the odds ratio (OR) of the number of lymph nodes examined (<12 vs ≥12) and exact 95% confidence intervals (CIs). Furthermore, the 5-year cancer specific survival (CSS) was calculated with Kaplan-Meier method, log-rank tests were used to compare the differences of CSS curves between patients with <12 and ≥12 lymph nodes examined. P<0.05 (two sides) was considered to be statistical significance. The statistical analyses were performed by using SPSS statistical software, version 20 (IBM Corp, Armonk, NY, USA).

## References

[1] Wolpin BM, Meyerhardt JA, Mamon HJ, Mayer RJ (2007). Adjuvant treatment of colorectal cancer. CA Cancer J Clin.

[2] Chen SL, Bilchik AJ (2006). More extensive nodal dissection improves survival for stages I to III of colon cancer: a population-based study. Ann Surg.

[3] Swanson RS, Compton CC, Stewart AK, Bland KI (2003). The prognosis of T3N0 colon cancer is dependent on the number of lymph nodes examined. Ann Surg Oncol.

[4] Bui L, Rempel E, Reeson D, Simunovic M (2006). Lymph node counts, rates of positive lymph nodes, and patient survival for colon cancer surgery in Ontario, Canada: a population-based study. J Surg Oncol.

[5] Nelson H, Petrelli N, Carlin A, Couture J, Fleshman J, Guillem J, Miedema B, Ota D, Sargent D (2001). Guidelines 2000 for colon and rectal cancer surgery. J Natl Cancer Inst.

[6] Otchy D, Hyman NH, Simmang C, Anthony T, Buie WD, Cataldo P, Church J, Cohen J, Dentsman F, Ellis CN, Kilkenny JW, Ko C, Moore R, Orsay C, Place R, Rafferty J (2004). Practice parameters for colon cancer. Dis Colon Rectum.

[7] Cone MM, Shoop KM, Rea JD, Lu KC, Herzig DO (2010). Ethnicity influences lymph node resection in colon cancer. J Gastrointest Surg.

[8] Johnson PM, Malatjalian D, Porter GA (2002). Adequacy of nodal harvest in colorectal cancer: a consecutive cohort study. J Gastrointest Surg.

[9] Bull AD, Biffin AH, Mella J, Radcliffe AG, Stamatakis JD, Steele RJ, Williams GT (1997). Colorectal cancer pathology reporting: a regional audit. J Clin Pathol.

[10] Scott KW, Grace RH, Gibbons P (1994). Five-year follow-up study of the fat clearance technique in colorectal carcinoma. Dis Colon Rectum.

[11] Stojadinovic A, Nissan A, Wainberg Z, Shen P, McCarter M, Protic M, Howard RS, Steele SR, Peoples GE, Bilchik A (2012). Time-dependent trends in lymph node yield and impact on adjuvant therapy decisions in colon cancer surgery: an international multi-institutional study. Ann Surg Oncol.

[12] Baxter NN, Ricciardi R, Simunovic M, Urbach DR, Virnig BA (2010). An evaluation of the relationship between lymph node number and staging in pT3 colon cancer using population-based data. Dis Colon Rectum.

[13] van Erning FN, Crolla RM, Rutten HJ, Beerepoot LV, van Krieken JH, Lemmens VE (2014). No change in lymph node positivity rate despite increased lymph node yield and improved survival in colon cancer. Eur J Cancer.

[14] Parsons HM, Tuttle TM, Kuntz KM, Begun JW, McGovern PM, Virnig BA (2011). Association between lymph node evaluation for colon cancer and node positivity over the past 20 years. JAMA.

[15] Markl B, Schaller T, Krammer I, Cacchi C, Arnholdt HM, Schenkirsch G, Kretsinger H, Anthuber M, Spatz H (2013). Methylene blue-assisted lymph node dissection technique is not associated with an increased detection of lymph node metastases in colorectal cancer. Mod Pathol.

[16] Ko CY, Chang JT, Chaudhry S, Kominski G (2002). Are high-volume surgeons and hospitals the most important predictors of in-hospital outcome for colon cancer resection?. Surgery.

[17] Ong ML, Schofield JB (2016). Assessment of lymph node involvement in colorectal cancer. World J Gastrointest Surg.

[18] Chang GJ, Rodriguez-Bigas MA, Skibber JM, Moyer VA (2007). Lymph node evaluation and survival after curative resection of colon cancer: systematic review. J Natl Cancer Inst.

[19] Prandi M, Lionetto R, Bini A, Francioni G, Accarpio G, Anfossi A, Ballario E, Becchi G, Bonilauri S, Carobbi A, Cavaliere P, Garcea D, Giuliani L, Morziani E, Mosca F, Mussa A (2002). Prognostic evaluation of stage B colon cancer patients is improved by an adequate lymphadenectomy: results of a secondary analysis of a large scale adjuvant trial. Ann Surg.

[20] Le Voyer TE, Sigurdson ER, Hanlon AL, Mayer RJ, Macdonald JS, Catalano PJ, Haller DG (2003). Colon cancer survival is associated with increasing number of lymph nodes analyzed: a secondary survey of intergroup trial INT-0089. J Clin Oncol.

[21] Sarli L, Bader G, Iusco D, Salvemini C, Mauro DD, Mazzeo A, Regina G, Roncoroni L (2005). Number of lymph nodes examined and prognosis of TNM stage II colorectal cancer. Eur J Cancer.

[22] Nash GM, Row D, Weiss A, Shia J, Guillem JG, Paty PB, Gonen M, Weiser MR, Temple LK, Fitzmaurice G, Wong WD (2011). A predictive model for lymph node yield in colon cancer resection specimens. Ann Surg.

[23] Mekenkamp LJ, van Krieken JH, Marijnen CA, van de Velde CJ, Nagtegaal ID (2009). Lymph node retrieval in rectal cancer is dependent on many factors--the role of the tumor, the patient, the surgeon, the radiotherapist, and the pathologist. Am J Surg Pathol.

[24] Hankey BF, Ries LA, Edwards BK (1999). The surveillance, epidemiology, and end results program: a national resource. Cancer Epidemiol Biomarkers Prev.

